# House Flies Are Underappreciated Yet Important Reservoirs and Vectors of Microbial Threats to Animal and Human Health

**DOI:** 10.3390/microorganisms11030583

**Published:** 2023-02-25

**Authors:** Dana Nayduch, Saraswoti Neupane, Victoria Pickens, Tanya Purvis, Cassandra Olds

**Affiliations:** 1 Arthropod-Borne Animal Diseases Research Unit, United States Department of Agriculture, Agricultural Research Service, 1515 College Avenue, Manhattan, KS 66502, USA; 2 Department of Entomology, Kansas State University, Manhattan, KS 66506, USA

**Keywords:** house fly, vector, bacterial pathogen, antimicrobial resistance, AMR, antibiotic resistance

## Abstract

House flies are well recognized as filth-associated organisms and public nuisances. House flies create sanitation issues when they bridge the gap between microbe-rich breeding environments and animal/human habitations. Numerous scientific surveys have demonstrated that house flies harbor bacterial pathogens that pose a threat to humans and animals. More extensive and informative surveys incorporating next-generation sequencing technologies have shown that house fly carriage of pathogens and harmful genetic elements, such as antimicrobial resistance genes, is more widespread and dangerous than previously thought. Further, there is a strong body of research confirming that flies not only harbor but also transmit viable, and presumably infectious, bacterial pathogens. Some pathogens replicate and persist in the fly, permitting prolonged shedding and dissemination. Finally, although the drivers still have yet to be firmly determined, the potential range of dissemination of flies and their associated pathogens can be extensive. Despite this evidence, the house flies’ role as reservoirs, disseminators, and true, yet facultative, vectors for pathogens have been greatly underestimated and underappreciated. In this review, we present key studies that bolster the house fly’s role both an important player in microbial ecology and population biology and as transmitters of microbial threats to animal and human health.

## 1. Introduction

**Figure 1:**
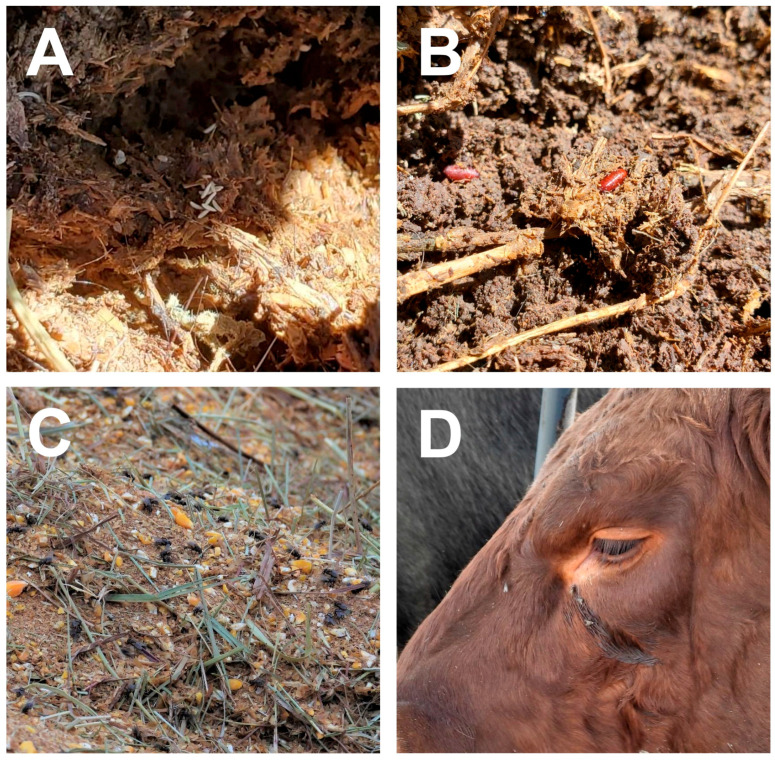
House flies associate with animals, their waste, and feed in livestock operations. House fly eggs (**A**) and pupae (**B**) in cattle manure. Adult house flies feeding on food in feed bunk (**C**) and cattle eye secretions (**D**). Photos by Victoria Pickens.

House flies (*Musca domestica* L.) are ubiquitous cosmopolitan pests occupying a variety of environmental niches on a global scale. Flies flourish in environments where they have access to microbe-rich substrates, which are essential for the development and fitness of the larval progeny [1,2]. Concentrated animal feeding operations (CAFOs) produce copious amounts of microbe-rich manure that comprises suitable larval developmental sites; as a result, fly populations can quickly reach nuisance levels in a short amount of time. Female house flies can lay over 100 eggs in a single batch, with over 500 in a lifetime (Figure 1A, [3]). Under optimal temperature conditions, larval development (Figure 1B) can be completed in under a week. Emerged adults are sexually receptive quickly and can mate successfully within a day of eclosion [4], and the first clutch of eggs can be laid less than two days after mating [3]. Alarmingly, resistance to pesticides, the mainstay of fly control for generations, is exceedingly high in house flies [5,6]. Typically, the aim of fly control in animal production systems has been to reduce population size and therefore nuisance activities, with significantly less focus being on limiting their potential to vector microbes.

Due to their mobile nature, adult house flies encounter microbes from disparate locations within a facility that would otherwise not interact. House flies are typically seen resting on physical structures (buildings, equipment, fencing, feed bunkers), feeding or inspecting animal feed and feed storage areas, and on the animals themselves (Figure 1C,D). Adult flies have broad trophic associations, consuming a variety of substrates that range from human and livestock food to their excreta. Female flies directly consume microbe-rich substrates such as manure and are possibly driven by opportunity during oviposition or perhaps a direct-yet-undetermined need for microbial nutrition [7]. While adult male flies tend to prefer carbohydrate-rich food sources, given a choice, females are attracted to protein-rich options [8]. The preference is most likely due to their anautogenous reproductive physiology, which requires protein for egg development (vitellogenesis). Female flies in agricultural settings are often seen feeding on proteinaceous secretions and excretions of animals (eye exudate, mucus, weeping wounds, blood, and manure) (Figure 1D). Not surprisingly, a greater abundance of bacteria is found in female than male flies, possibly due to their propensity for microbe-rich environments [9,10]. The greatest threat posed by house flies is their ability to acquire and move pathogens from low-risk areas such as manure to high-risk areas such as feed (both human and animal). Being both zoophilic and synanthropic, house flies bridge the gap between filth and animals (including humans) both within environmental niches and across them. At a minimum, house flies serve as reservoirs and disseminators of microbes in the environment; therefore, they are integral to microbial ecology from both a small scale, such as within a farm, to larger landscape scales, such as CAFOs and surrounding communities, as well as urban locales where access to waste (e.g., dumpsters) exists. In this review, evidence highlighting the role of flies as reservoirs and disseminators of microbes is presented and the implication for animal and human health discussed.

## 2. Adult House Fly Associations with Bacteria and Vector Potential

When adult house flies interact with microbe-rich substrates, they become contaminated with bacteria on their external surfaces. Bacteria on the fly’s mouthparts, tarsi, wings, and body becomes dislodged from and transferred to the locations flies subsequently visit by direct contact (i.e., mechanical transfer), feeding (i.e., contaminated mouthparts), and/or grooming (i.e., removal and disposal) [11,12]. Flying has been shown to negatively impact viability of bacteria on flies presumably by hastening drying [11]; however, bacteria located within crevices of the house fly external anatomy may have increased protection that enhances persistence as shown for *Escherichia coli* O157:H7, which survived on the fly body up to 13 d after bacterial exposure [13]. Mechanical transmission from the external surfaces likely applies to both female and male flies that both frequent and interact with septic substrates.

Flies also carry bacteria internally within their gut, acquired either indirectly when auto-grooming or directly by intentionally ingesting the microbe-rich substrate [14]. Ingestion of bacteria has been more often observed in female flies, who visit and inspect substrates such as manure and dumpster sludge as potential oviposition sites [7]. After ingestion, bacteria may be stored in the crop (a diverticulated storage area of the insect foregut where ingested substances are held prior to digestion in the midgut), from where they can be regurgitated, or the midgut, where they face hostile digestive and defensive processes. Bacteria that survive these midgut challenges may pass through the alimentary canal and are released to the environment via defecation. Recent studies showed that internal bacterial communities of individual house flies are comprised of up to 400 bacterial taxa [15,16,17], while other studies that employ next generation sequencing of whole flies (accounting for internal and external bacteria) have revealed that an individual fly can carry >1600 operational taxonomic units [18,19,20].

Demonstrating the ability of house flies to act as bacterial vectors is key in understanding their true risk to animal and human health. Indirect evidence of house fly vector potential was supported when the presence of house flies was associated with a 10-fold increase in the number of fecal coliforms (*Escherichia coli* and *Klebsiella* spp.) on steam-flaked corn [21]. Other studies have implicated house flies as bacterial vectors in the field by identifying bacterial genotypes shared between the environment and house flies caught in the immediate area, thereby deducing sources of acquisition and connecting to subsequent fly harborage and dissemination [22,23,24]. More direct evidence of house fly bacterial transmission has been shown via bioassays where flies acquired *Salmonella enterica* Serovar Typhimurium from inoculated manure, subsequently transmitting the pathogen to cantaloupe [25]. Although it likely occurs naturally at a high frequency, especially in animal production settings, experiments directly demonstrating transmission of bacteria from house flies to animals or people are sparse. However, one study demonstrated successful transmission of pathogenic *E. coli* O157:H7 to naïve calves [26], and house flies were also implicated in an outbreak of *E. coli* O157:H7 in people in Japan [27].

“Mechanical transmission” of bacteria by flies has been used to describe transmission of nonreplicating microbes from fly surfaces, vomit, and feces, while the term “bio-enhanced transmission” was suggested for *E. coli* that replicated in the anterior alimentary canal of house flies before transmission [28]. However, if conditions exist where bacteria are ingested, proliferate, and survive passage through the digestive system, then house flies are indeed, by definition, facultative biological vectors of those microbes. House flies’ effectiveness as vectors depends on several factors including bacterial viability. Several studies have investigated the “fate” of bacteria ingested by house flies, mainly through assays that feed flies a set amount of pure culture of a single bacterial species and monitor bacterial abundance and/or excretion over time. Some bacterial species were only transient residents within the fly digestive system that exhibited short-term viability. For example, the Gram-positive cocci, *Staphylococcus aureus* [29] and *Streptococcus pyogenes* [30], did not proliferate or persist for long periods in the house fly gut, and microscopic observations of the bacteria in the gut indicated these microbes were rapidly lysed (over 75% of ingested inoculum is lost by 6 h in each species), likely by lysozymes or other antimicrobial and/or digestive enzymes, thereby precluding transmission via defecation. A maximum of 30 colony-forming units of *S. pyogenes* was shed by only ~23% of flies in the study [30], indicating that house flies are not important vectors or reservoirs for this bacterial species. However, flies shed large amounts of *S. aureus*, likely through regurgitation from the crop, within a short time window post-ingestion (PI) [29]. Thus, although house flies may only serve as short-range transmitters of *S. aureus*, this phenomenon still could be functionally relevant where animals and sources of bacteria are in close proximity, such as dairy cattle operations.

In contrast, other studies have shown that some bacteria have extended survival or even propagated within the house fly gut. GFP-expressing *Enterococcus faecalis* proliferated in a crop of house flies from exposure through 48 h, and bacterial abundance remained constant until the end of the experiment (96 h) [31]. *Aeromonas caviae* proliferated in house flies for up to 2 d PI, persisted for up to 8 d PI, and were shed for up to 3 d PI [32]. Both *Pseudomonas aeruginosa* [33] and *Salmonella typhimurium* [34] proliferated in the house fly digestive tract (maximum of 115% and 600% increase from initial dose, respectively), showing temporal dynamics that indicated apparent attrition that possibly was mediated via house fly excretion or gut defenses, followed by recovery, proliferation, and multiple shedding events. Bacterial persistence, proliferation, and excretion into the environment bolsters biological vectoring of these pathogens by the house fly, and at the very least indicates an increased risk for transmission and dissemination.

Other bacterial species such *E. coli*, *Campylobacter jejuni*, and *Aeromonas hydrophila* seem to have intermediate outcomes within the gut of flies. Some strains of *E. coli* survived and persisted in the fly gut but did not seem to proliferate [28,35,36,37]; however, viable bacteria have been recovered from excreta, indicating a transmission risk still exists. Flies fed *C. jejuni* maintained viable bacteria for up to 24 h, but no culturable bacteria were recovered from excreta past 4 h PI [38]. *Aeromonas hydrophila* proliferated in flies (up to 1000× initial dose was ingested), though only for a short window of time (2 h PI; [39]). Viable bacteria were recovered from regurgitant for up to 2 h PI, though not from feces, indicating the bacteria were likely proliferating in the house fly crop, but were destroyed when they entered the gut. Thus, like *S. aureus*, flies may be short-term and short-distance mechanical vectors for both *E. coli* and *C. jejuni*, which can prove relevant under crowded conditions that typically exist in CAFOs such as feedlots (for *E. coli*) and poultry houses (for both species). Similarly, flies may be short-range transmitters of *A. hydrophila* from sources such as waste dumpsters to nearby homes or restaurants.

## 3. Wild House Flies Are Potential Threats to Animal and Human Health

Global surveys demonstrated that adult house flies from urban and agricultural settings possess >200 pathogenic bacterial taxa including key species important to human and animal health such as *Salmonella* spp. [14,40,41,42,43,44,45,46], pathogenic strains of *E. coli* [22,47,48,49,50,51,52], *Campylobacter* spp. [49,50,53,54,55,56], *Aeromonas cavie* [57], and bacterial pathogens causing bovine respiratory disease *Mannheimia haemolytica*, *Pasteurella multocida*, *Histophilus somni* [58]. Culture-based and more recently (i.e., within the past 20 years) molecular-based surveys have provided even more extensive lists of pathogens carried by flies, which have already been well summarized in other studies or reviews [14,15,16,17,18,59,60,61,62,63,64] and will not be recapitulated here. As carriers of many pathogenic bacteria, adult house flies are undoubtedly key players in food security, specifically in pre- and post-harvest food safety across a variety of food animal species [42,65,66].

## 4. House Flies as Reservoirs and Disseminators of Antimicrobial Resistance

Widespread and/or overuse of antibiotics, particularly in the production of animals for food, often selects for the emergence of antimicrobial resistance (AMR) in both animal and human pathogens [67,68,69]. Broad-spectrum antibiotics such as tetracyclines, florfenicol, enrofloxacin, and ceftiofur [70,71] are commonly used for metaphylaxis to prevent or mitigate suspected infection and disease. Although now banned in many countries, the mass administration of antibiotics as growth promoters still occurs (reviewed by [72]). As a result of this indiscriminate overuse, food animals maintain a plethora of drug-resistant bacterial species, which they shed in their excreta that is subsequently accessed by house flies.

House flies collected from animal production facilities (Table S1; [10,22,43,45,73,74,75,76,77,78,79,80,81,82,83,84,85,86,87,88] and urban environments [10,63,84] exhibit a wide diversity of AMR bacteria including potential human and animal pathogenic species. Multi-drug-resistant bacteria have also been isolated from these flies including *Staphylococcus* spp. isolated with multiple antibiotic-resistant genes (ARGs; *msrA*, *msrB*, *tetK*, *tetM*, *tetL*, *ermC*, and *aad6*) exhibiting multidrug resistance (oxacillin, penicillin, erythromycin, clindamycin, tetracycline, ciprofloxacin, gentamicin, and chloramphenicol) phenotype [63]. *Providencia* spp. *Enterobacter* spp. and *E. coli* from house flies with colistin resistance genes (*mcr-1*, *mcr-2*, *mcr-3*) were highly prevalent in a variety of ecological niches including animal production facilities [45,82,89]. In addition, a high prevalence of *E.coli* with several extended-spectrum beta-lactamase genes (bla_CTX-M_, bla_TEM_) and tetracycline resistance genes (*tetA*, *tetB*, *tetD*, *tetM*) are highly prevalent in house flies associated with animal agriculture [77,78,82].

Not only do house flies harbor AMR bacteria, but, alarmingly, house flies can facilitate the transfer of AMR elements between bacterial species in their gut (reviewed in [24,31,61,90,91,92]. Horizontal gene transfer via plasmid transfer or phage transduction has been demonstrated to occur in the house fly gut. Plasmid-encoded chloramphenicol resistance genes were horizontally transferred from donor to recipient bacteria inside the gut of the house fly, while bacteriophage containing Shiga toxin genes (*stx1*) were transduced to recipient *E. coli* [90]. Plasmids carrying cephalosporin and tetracycline resistance genes were transferred horizontally from donor to recipient bacteria in the house fly gut [92]. Interestingly, in addition to the intended recipient *E. coli* strain, *Achromobacter* spp. and *Pseudomonas fluorescens* present in the gut also acquired the resistance element [92]. Gene transfer events can happen very quickly, with conjugative plasmids carrying tetracycline resistance being shared among enterococci within 24 h of exposure [91]. In the environment, house flies visit numerous sources of microbes, facilitating interactions between bacteria collected from physically separated habitats which otherwise would not interact. As such, they contribute significantly to the dissemination of AMR bacteria and their resistance genes across habitats and ecological niches.

## 5. Unique Modes of Bacteria Dispersal from House Flies

In addition to the mechanical pathogen transmission and dissemination of bacteria by flies, described above, some methods used to control house flies have been shown to lead to unintentional dispersal of bacteria and other microbes. Electrocuting insect traps (EIT) have been used as an alternative to pesticides for house flies, especially in areas such as restaurants and human dwellings. Such traps cause fly body particulates to scatter around the trap [93], contaminating nearby areas, or aerosolize as a respirable particle size after electrocution [94,95]. Microbes associated with insect debris and microbial aerosols could be distributed by the electrocution of flies in both high- and low-voltage light traps [96], with a majority of aerosolized microbes at a size capable of penetrating the lung alveoli.

Investigations by [97] revealed that ingested *Serratia marcescens* remains viable in house flies for up to 5 weeks following electrocution. Post-fly death bacterial dissemination could also occur with other control methods which kill house flies without collecting the carcass. Considering that many insecticides are not immediately lethal or are slow acting, house flies may be given enough time to reach another area of a facility before succumbing to the toxic effects. In some cases, they might die in or near animal feed, where they can be accidentally consumed. Since the bacterial load of the whole fly is often higher than that of the feces or regurgitant [29,39], in animal species prone to eating insects such as poultry, dead flies might even result in intentional consumption by the animal, resulting in higher transmission risk. Viral infection through the consumption of deceased flies has already been demonstrated in the case of Aujeszky’s virus in swine [98], as well as the bacterial infection of chickens with *Salmonella enterica* serovar Enteritidis [66]. Further research is needed to estimate the rates at which consumption of house flies occurs in livestock facilities, as well as the survivability of pathogenic microorganisms in post-mortem house flies, to assess the potential for animal infection by contaminated house flies if ingested.

## 6. House Fly Movement and the Implication for Pathogen Transmission

The highly mobile nature of the house fly both within and from animal rearing facilities is key to its threat as a vector. Changing location and quality of nutritional and ovipositional resources is likely a motivator for flies to move from one place to another, with the nature of these drivers varying over the lifespan of the fly. With fly movement comes the movement of their associated bacteria, so understanding fly movement both within and dispersal from animal facilities is key in evaluating the risks posed to animal and human health. How far and why flies fly have been key questions asked by researchers since their identification as vectors of pathogens. While an individual fly’s movement patterns are more complex than we currently understand, some variables driving movement have been identified. For example, fly sex may underlie differences in movement and activity. A newly emerged adult female will first move in an attempt to locate a source of food high in sugar, later switching and seeking out food high in protein, which is important for vitellogenesis [99]. During the latter stages of vitellogenesis, she will eschew the attention of males, possibly even relocating to a different food source to avoid male attention [99]. Fly sexes show different diurnal activity, with male flies being active earlier in the day during cooler temperatures [100].

**Figure 2:**
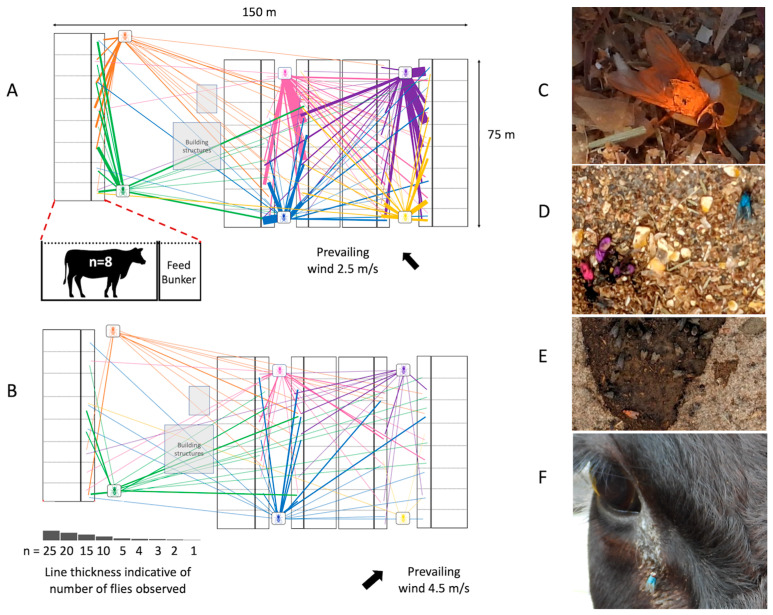
House fly movement within a concentrated animal feeding operation. Location of colored flies observed 1 h (**A**) and 24 h (**B**) after release. Flies were predominantly observed in feed bunkers containing animal feed (**C**,**D**) though also on manure (**E**) and animals (**F**). Photos by Cassandra Olds.

Fly movement on a small scale can be quantified and observed using mark–release–recapture studies. In a small demonstration, approximately 4000 flies were collected by sweep netting at a small cattle feedlot (under 350 head in a 11,250 m^2^ area), and 600–700 flies were colored in 1 of 6 colors with mica powder (Olds, unpublished data). Flies were released and their location within the feedlot monitored (Figure 2). While many flies stayed close to their release site (within the feed bunker corridor), flies of all colors were observed in all areas in as little as 1 h after release. Flies remain marked for 36 h, after which point no marked flies could be observed. Although this experiment was performed in a relatively small location, it can be reasonably assumed that similar patterns of movement would occur in larger facilities.

**Figure 3:**
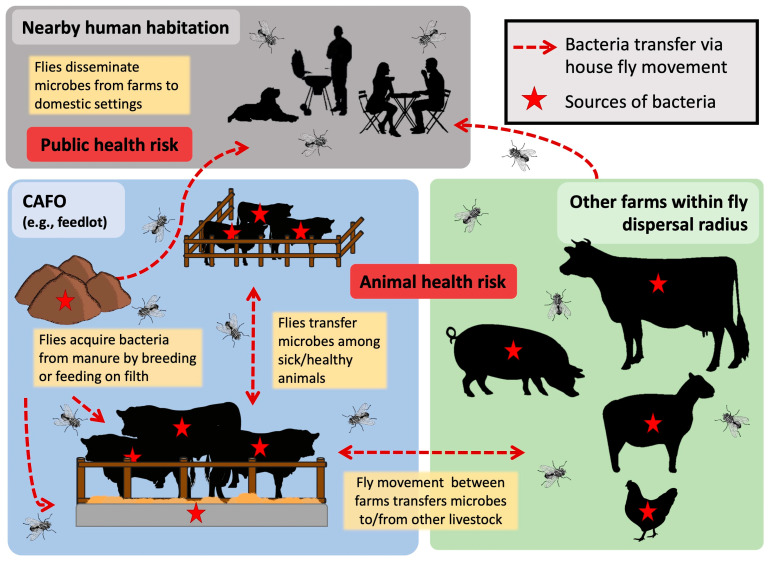
House flies pose a risk to human and animal health by disseminating and transmitting microbes. House flies acquire microbes such as bacteria from sources such as animals and their waste, and disseminate within and among farms, as well as to nearby areas of human habitation.

As outlined above, in animal feeding operations, flies have access to sources of microbes (from animals and their waste) and move about freely among these sources and the animals’ food, water, and environment (Figure 3). The propensity of flies to freely move between sanitary and unsanitary areas in animal production operations has important implications for the movement of pathogenic, and potentially antimicrobial-resistant microbes. Flies are regularly found to have bacteria associated with animal waste such as fecal coliforms and enteric pathogens that they likely acquire from manure. Flies also have been implicated in the movement of bacterial pathogens of bovine respiratory disease (BRD) because they carried the causative agents—*M. haemolytica*, *P. multocida, and H. somni*—around pen areas housing cattle with apparent BRD symptoms [58]. Whether flies acquired those bacteria from the sick animals in the pen or were involved in the transmission was not determined. Nonetheless, sanitation or disease-limiting efforts such as cleaning out pens and piling waste, separating sick animals from well ones, or separating types of animals in a multi-species production setting can be easily undermined if fly control is not effective.

Factors driving house fly dispersal are complex and sometimes ambiguous, but as with insects, most individuals in the population likely remain in their natal site, with only a few moving outside of that to a new environment (reviewed by [101]). Multiple catch-and-release studies confirm that most house flies stay within the ‘home’ environment, with fewer flies being caught further out [102,103,104,105,106]. However, despite the persistent presence of excellent nutritional and ovipositional resources, flies still readily disperse from animal production facilities, as evidenced most dramatically by fly nuisance lawsuits filed against livestock producers [106,107,108]. In addition to nuisance, the movement of flies away from animal production to nearby human habitation poses additional risks of contamination and disease transmission (Figure 3). House flies collected from urban areas shared potential pathogenic bacterial taxa *Corynebacterium*, *Clostridium*, and *Escherichia-Shigella* as well as rumen-associated bacterial taxa *Romboutsia*, Ruminococcaceae, and Lachnospiraceae, which were probably acquired from a nearby agricultural farm [17], as cattle excretions and manure are known sources of these taxa [109,110]. Several mark–release–recapture studies have shown that when flies do disperse, they can travel vast distances. Reported flight distances vary significantly (7–32 km) depending on the location, vegetation, landscape barriers, and drivers for dispersion [103,104,105,111,112]. Flies can easily enter motorized vehicles, and are either released along the way or once the final destination is reached, significantly increasing dispersal potential.

## 7. Conclusions

House flies are trophically associated with microbe-rich substrates in all habitats they occupy, from manure and waste generated by CAFOs to urban dumpsters. These environments are teeming with microbes, many of which are pathogenic and/or carry antimicrobial resistance elements. Flies have been demonstrated to harbor and disseminate viable microbial pathogens, some of which have been shown to proliferate in the fly, thereby increasing their vector potential. Furthermore, house flies can facilitate the transfer of antimicrobial resistance and other genetic elements among the bacteria contained within their gut. As gregarious and indiscriminate feeders, house flies bridge the gap between filth or other sources of bacteria (manure, feed waste, sick animals, etc.) and healthy animals or areas of human habitation. It is abundantly clear that house flies are much more than just a nuisance problem in food animal production settings. Without the use of proper integrated pest management strategies, practices of removing or distancing filth such as manure and sick animals from clean areas (healthy animals and their pens) become useless when open access to these niches by flies exists. Fly control should be paramount in any hygiene plan in agricultural or residential settings, the foundation of which should be the identification and removal of larval habitats that are not only sources of bacteria but also breeding grounds for the flies that carry the bacteria. Thus, sanitation effectively removes both sources of dangerous microbes as well as larval development sites, reducing fly populations and bacterial acquisition risk. House flies have been largely underappreciated not only in their role in the microbial ecology of the bacteria they harbor but also in their ability to act as competent vectors of human and animal pathogens. However, while flies may pose a threat to animal and human health, surveying house fly microbial communities has potential utility in allowing us to monitor microbial threats in the environment.

## Data Availability

The data that support the findings of this study are included in the article and supplementary materials.

## Supplementary Materials

The following supporting information can be downloaded at: https://www.mdpi.com/article/10.3390/microorganisms11030583/s1, Table S1: Bacteria with antibiotic resistance phenotypes isolated from house flies associated with food and companion animal facilities.
